# Phenotypic Variation in Water-Use Efficiency, Heat Tolerance, and Carbon Isotope Discrimination Across Canadian Spring Wheat Cultivars Under Climate Stress

**DOI:** 10.3390/plants15131958

**Published:** 2026-06-25

**Authors:** Ludovic Joseph Anatole Capo-chichi, Scott X. Chang, Pierre Hucl, Mazen Aljarrah, Jennifer Zantinge, Michael Holtz, Ammar Elakhdar, Muhammad Iqbal, Guillermo Hernandez-Ramirez

**Affiliations:** 1 Department of Renewable Resources, Faculty of Agricultural, Life and Environmental Sciences, 751 General Services Building, University of Alberta, Edmonton, AB T6G 2H1, Canada; 2 Crop Development Centre, Room 4D36 Agriculture Building, University of Saskatchewan, 51 Campus Drive, Saskatoon, SK S7N 5A8, Canada; 3 Western Crop Innovations, 5030–50 Street, Lacombe, AB T4L 1W8, Canada; 4 Field Crops Research Institute, Agricultural Research Centre, Giza 12619, Egypt; 5 Department of Agriculture, Food and Nutritional Science, 4-10 Agriculture-Forestry Centre, University of Alberta, Edmonton, AB T6G 2P5, Canada

**Keywords:** phenotypic diversity, spring wheat, stable isotope, abiotic stress tolerance

## Abstract

Understanding phenotypic variation in traits associated with drought and heat tolerance is essential for developing climate-resilient spring wheat cultivars under increasingly variable environmental conditions. To evaluate phenotypic and physiological variation in water-use efficiency (WUE), carbon isotope discrimination (δ^13^C), and heat tolerance, 198 Canadian spring wheat cultivars representing diverse breeding backgrounds were assessed under controlled drought and high-temperature conditions. Traits measured included whole-plant water-use efficiency (WUE_WP_), carbon isotope composition (δ^13^C), biomass accumulation, water use per plant, and chlorophyll fluorescence across six developmental stages. Whole-plant WUE ranged from 3.07 to 7.81 g L^−1^, while δ^13^C values ranged from −24.06‰ to −29.33‰. Biomass accumulation and water use were strongly positively correlated (r = 0.94, *p* < 0.001), indicating that greater biomass production was associated with increased water consumption. In contrast, the relationship between WUE_WP_ and δ^13^C was weak (r = −0.09), suggesting that δ^13^C alone may not be a reliable proxy for WUE_WP_ under combined drought and heat stress conditions. Phenotypic diversity across the cultivar panel was relatively low to moderate (Shannon diversity index, *H^′^* = 1.88–2.62), indicating limited adaptive capacity within the evaluated germplasm. Principal component analysis explained 76.6% of the total variation and effectively differentiated cultivar responses to stress. Chlorophyll fluorescence, particularly the maximum quantum efficiency of PSII photochemistry (F_V_/F_M_), was highly sensitive to stress-induced reductions in photosynthetic performance. Measurements obtained during reproductive drought and heat stress stages showed stronger associations with biomass, water use, WUE_WP_, and δ^13^C than measurements collected during non-stress periods, indicating that F_V_/F_M_ can be a reliable physiological indicator for screening drought and heat tolerance. Overall, the results revealed detectable phenotypic variation but relatively modest diversity and generally weak to moderate trait associations, highlighting the potential value of incorporating diverse germplasm and integrated phenotyping approaches to improve climate resilience in Canadian spring wheat.

## 1. Introduction

Wheat (*Triticum aestivum* L.) is the largest cereal crop in the Canadian Prairies; however, seasonal variability in moisture and temperature is a major constraint on plant development and productivity, often resulting in significant yield losses [1]. Climate projections by Environment Canada indicate an increasing drought frequency, rising temperatures, and reduced water availability across the region, posing significant risks to wheat productivity and long-term sustainability [2]. In recent years, producers have frequently experienced severe moisture deficits during the growing season, resulting in recurrent drought and heat stress. These conditions reduce yield and grain quality, and in extreme cases, can lead to partial or complete crop failure, thereby reducing farm profitability [3]. Data from Statistics Canada indicate that total wheat production declined by 38.5% due to moisture deficits, particularly during the 2021 and 2023 growing seasons [4]. Zhao et al. reported an average wheat yield loss of 50–60% under water-limited conditions [5]. High temperatures further exacerbate yield losses by inhibiting photosynthesis, accelerating chlorophyll degradation, and disrupting key physiological processes, resulting in cellular damage [6]. Temperature increases of only 3–4 °C above the optimum during grain filling can reduce wheat yield by 10–50%, depending on the cultivar [7,8]. These challenges underscore the urgent need to develop high-yielding, resilient wheat cultivars with enhanced drought and heat tolerance.

Crop water use efficiency (WUE) is a key trait for sustaining productivity in water-limited environments, where crop performance depends on the efficient use of available water resources [9]. WUE describes how efficiently a plant converts water into biomass or grain yield by balancing carbon assimilation with water loss through transpiration [10]. Enhancing WUE in spring wheat is therefore a central objective for improving resilience, stabilizing yield, and maintaining production under increasingly variable climatic conditions. Despite its importance, accurate quantification of WUE remains challenging. The trait integrates multiple physiological and physical processes, including photosynthesis, stomatal conductance, and evaporative demand, that vary spatially and temporally. In addition, WUE is strongly influenced by environmental conditions such as soil moisture, temperature, and atmospheric demand, complicating both phenotypic assessment and genetic dissection [11,12].

Various direct and indirect methods have been developed to estimate WUE [13,14], yet each approach has limitations in accuracy, scale dependency, and data requirements, leading to inconsistent results across environments. Traditionally, WUE has been estimated from leaf-level gas exchange measurements of photosynthesis and transpiration, assuming these are representative of whole-plant performance [15,16,17]. In practice, WUE can be assessed at multiple scales, ranging from the leaf and whole-plant levels to population or field scales (biomass-based WUE), each capturing different physiological and ecological aspects of plant water use.

At the leaf level, several physiological parameters have been proposed as indirect indicators of WUE and drought tolerance; among these, carbon isotope discrimination (δ^13^C) is particularly promising. δ^13^C provides a rapid and integrative measure of WUE by quantifying the ratio of ^13^C to ^12^C in plant tissue relative to atmospheric CO_2_ [18,19,20]. It reflects the balance between carbon assimilation and stomatal conductance, making it a reliable indicator of a plant’s capacity to use water efficiently. In wheat breeding programs, δ^13^C has been effectively used to identify genotypes with superior WUE and yield potential under drought conditions [21,22,23,24,25]. Under non-limiting water conditions, positive correlations between δ^13^C, grain yield, and biological yield have also been reported [26].

The ratio of mesophyll to stomatal conductance (g_m_/g_s_) represents a key determinant of CO_2_ uptake efficiency, while respiration rate was identified as another major factor influencing WUE because increased respiration reduces net carbon assimilation [27,28]. Other physiological traits have also been suggested as useful indicators for screening crops for drought tolerance and WUE [29]. Leaf-level WUE is a complex trait influenced by multiple physiological processes affecting photosynthesis and transpiration [28].

At the whole-plant or population level, WUE is defined as the ratio of biomass or grain yield produced per unit of water consumed [30,31,32,33]. This parameter is affected by genetic factors that influence either biomass production or transpiration efficiency. Substantial phenotypic variation for WUE has been reported in several crops, including barley (*Hordeum vulgare* L.) [19,34,35], grapevine (*Vitis vinifera* L.) [36,37], cowpea (*Vigna unguiculata* (L.) Walp.) [38], peanut (*Arachis hypogaea* L.) [39], sorghum (*Sorghum bicolor* (L.) Moench) [40], soybean (*Glycine max* (L.) Merr.) [41,42], cotton (*Gossypium hirsutum* L.) [43,44], and wheat [45,46,47].

Phenotypic diversity underpins progress in improving WUE because it enables the identification and selection of genotypes with favorable combinations of water-use traits [48]. Conventional breeding has traditionally relied on field-based assessments of biomass production and grain yield under contrasting soil moisture conditions, while recent advances in high-throughput phenotyping have enabled more precise and large-scale evaluation of WUE-related traits across diverse germplasm populations [49]. Despite these advances, important knowledge gaps remain regarding the physiological and genetic basis of WUE and stress adaptation in wheat. Most previous studies investigating WUE and δ^13^C have focused on relatively small genotype collections, specific breeding populations, or individual physiological traits, often under either drought or heat stress alone. Consequently, the extent to which WUE, δ^13^C, photosynthetic performance, biomass production, and water use are related under combined drought and heat stress remains poorly understood. In addition, limited information is available on the magnitude of phenotypic variation for these traits within Canadian spring wheat germplasm, particularly across cultivars developed by different breeding programs over more than a century of cultivar development.

This lack of comprehensive physiological characterization limits the identification of superior germplasm and trait combinations that could be used to improve climate resilience in wheat breeding programs. A better understanding of the diversity and interrelationships among WUE, δ^13^C, chlorophyll fluorescence traits, biomass production, and water use is needed to determine their potential value as selection criteria for improving adaptation to increasingly frequent drought and heat events. Therefore, a comprehensive evaluation of a large and diverse panel of Canadian spring wheat cultivars is required to address these knowledge gaps and provide insights into the physiological mechanisms underlying stress tolerance.

Accordingly, the objectives of this study were to: (a) assess the phenotypic diversity and relationships among whole-plant water-use efficiency (WUE_WP_), δ^13^C, and related physiological traits associated with drought and heat tolerance in Canadian spring wheat cultivars registered between 1905 and 2018; and (b) quantify the extent of phenotypic differentiation among breeding programs.

## 2. Results

### 2.1. Variation in WUE_WP_, δ^13^C, Biomass Accumulation and Water Use per Plant

Substantial phenotypic variation was observed among the wheat cultivars for δ^13^C, WUE_WP_, biomass accumulation, and water use under combined drought and heat stress (Table 1). In Growth Chamber Run 1, δ^13^C ranged from −29.33‰ to −24.28‰, WUE_WP_ from 3.07 to 5.71 g L^−1^, biomass from 9.50 to 23.85 g plant^−1^, and water use from 2.27 to 5.04 L plant^−1^. Significant cultivar effects were detected for WUE_WP_, biomass, and water use (*p* = 0.0003; *p* < 0.001; *p* = 0.0269, respectively). In Growth Chamber Run 2, δ^13^C ranged from −28.16‰ to −24.06‰, WUE_WP_ from 3.11 to 7.81 g L^−1^, biomass from 24.80 to 50.60 g plant^−1^, and water use from 3.17 to 12.34 L plant^−1^. Significant cultivar effects were observed for WUE_WP_ and biomass (*p* < 0.001), whereas water use was not significant (*p* = 0.34). Across both runs, δ^13^C ranged from −29.33‰ to −24.06‰, WUE_WP_ from 3.07 to 7.81 g L^−1^, biomass from 9.50 to 50.60 g plant^−1^, and water use from 2.27 to 12.34 L plant^−1^. Cultivar effects were significant for biomass and water use (*p* < 0.001 and *p* < 0.0001, respectively), while WUE_WP_ showed a marginal cultivar effect (*p* = 0.0554), highlighting considerable phenotypic variation among cultivars for traits related to productivity and water use.

Variation in biomass accumulation and water use was considerably greater than variation in δ^13^C and WUE_WP_, suggesting that cultivar responses to combined drought and heat stress were expressed primarily through differences in growth and water consumption rather than large shifts in carbon isotope composition. These findings indicate the existence of contrasting physiological strategies among Canadian spring wheat cultivars for coping with water-limited and high-temperature conditions.

**Figure 1:**
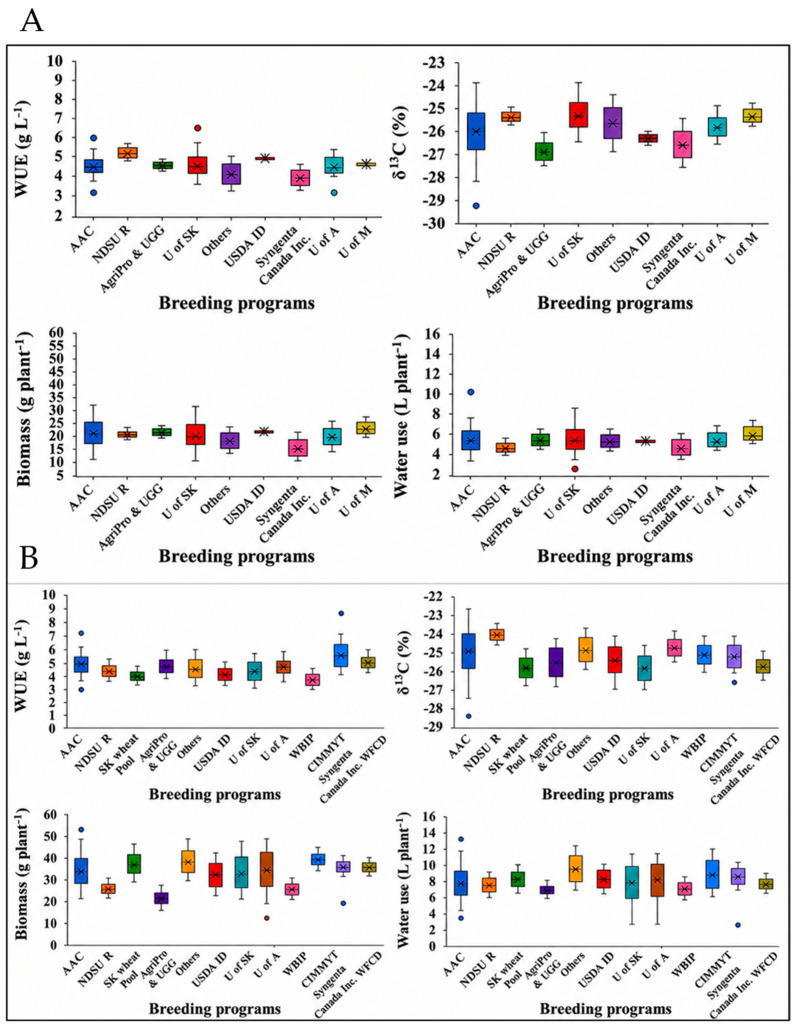
Variation in WUE_WP_, δ^13^C, water use per plant, and biomass accumulation among 198 Canadian spring wheat cultivars from multiple breeding programs. Cultivars were evaluated in two separate growth-chamber runs due to space limitations, with Group (**A**) (Growth Chamber Run 1) and Group (**B**) (Growth Chamber Run 2) representing cultivars assessed in the first and second runs, respectively (n = 99 cultivars per run). AAFC: Agriculture and Agri-Food Canada; U of A: University of Alberta; U of SK: University of Saskatchewan; U of M: University of Manitoba; WFGD: Western Feed Grain Development Inc.; CIMMYT: International Maize and Wheat Improvement Center; NDSU: North Dakota State University; USDA ID: United States Department of Agriculture; WPB/P: Wiersum Plant Breeding/Plantomar; AgriPro & UGG: AgriPro/United Grain Growers.

Differences in trait variability were also evident among breeding programs (Figure 1). Biomass accumulation and water use generally exhibited greater variation than WUE_WP_ and δ^13^C, indicating stronger divergence in growth and water-use characteristics than in intrinsic water-use efficiency. Breeding programs such as AAFC, AgriPro, UGG, Syngenta Canada, and the University of Manitoba tended to show broader distributions for one or more traits, whereas NDSU and WFGD generally exhibited narrower distributions and more uniform responses. Programs displaying greater variation in biomass and water use also tended to exhibit broader phenotypic responses to drought and heat stress, suggesting differences in the extent of phenotypic and physiological diversity maintained among Canadian spring wheat breeding programs.

**Figure 2:**
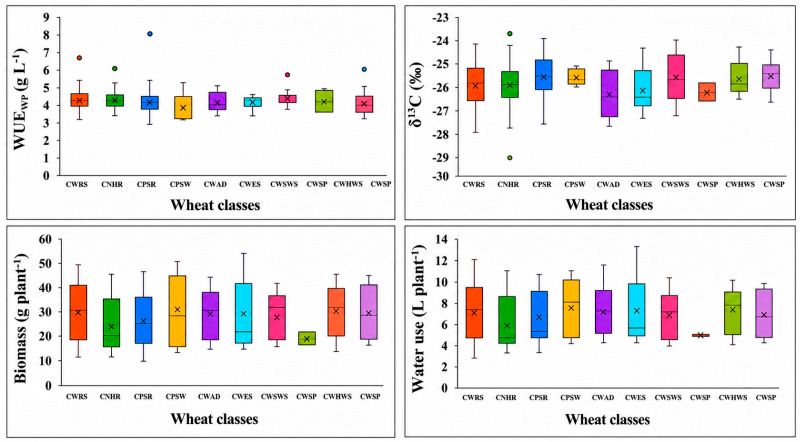
Phenotypic diversity for WUE_WP_, δ^13^C, whole plant biomass, and whole plant water use among 198 Canadian spring wheat cultivars derived from various wheat classes. CNHR: Canada Northern Hard Red; CPSR: Canada Prairie Spring Red; CPSW: Canada Prairie Spring White; CWAD: Canada Western Amber Durum; CWES: Canada Western Extra Strong; CWHWS: Canada Western Hard White Spring; CWRS: Canada Western Red Spring; CWSP: Canada Western Special Purpose; CWSWS: Canada Western Soft White Spring.

Substantial variation was observed among wheat classes for WUE_WP_, δ^13^C, biomass accumulation, and water use under combined drought and heat stress (Figure 2). Overall, biomass accumulation and water use exhibited greater variation than WUE_WP_ and δ^13^C, indicating that wheat classes differed more in growth performance and water consumption than in intrinsic water-use efficiency. Classes such as CWRS, CPSW, CWES, and CWHWS generally showed broader distributions for biomass and water use, whereas CWSP exhibited lower and more uniform values. In contrast, WUE_WP_ and δ^13^C were comparatively stable across classes, suggesting more consistent physiological responses.

The Shannon–Weaver diversity index (*H′*) and coefficient of variation (CV) further confirmed substantial phenotypic diversity among cultivars (Table 1). Diversity was generally greater in Growth Chamber Run 2 than in Growth Chamber Run 1, particularly for WUE_WP_, biomass accumulation, and water use. Across both runs, δ^13^C exhibited the highest diversity (*H′* = 2.61), followed by WUE_WP_ and biomass accumulation (*H′* = 2.52), while water use showed comparatively lower diversity (*H′* = 1.98). These results indicate considerable diversity for physiological and growth-related traits associated with adaptation to drought and heat stress.

Patterns of variability were consistent with the diversity analysis. Across runs, water use exhibited the highest CV values (11.41–19.87%), followed by biomass accumulation (12.13–13.48%), whereas WUE_WP_ showed the lowest variability (9.48–11.98%). Overall, cultivar differences were expressed more strongly through biomass production and water-use patterns than through water-use efficiency, while δ^13^C maintained consistently high phenotypic diversity across runs.

### 2.2. Relationship Among WUE_WP_, δ^13^C, Biomass, Water Use per Plant, and Maximum Quantum Efficiency of Photosystem II (F_V_/F_M_) Under Non-Stress and Stress Conditions

**Figure 3:**
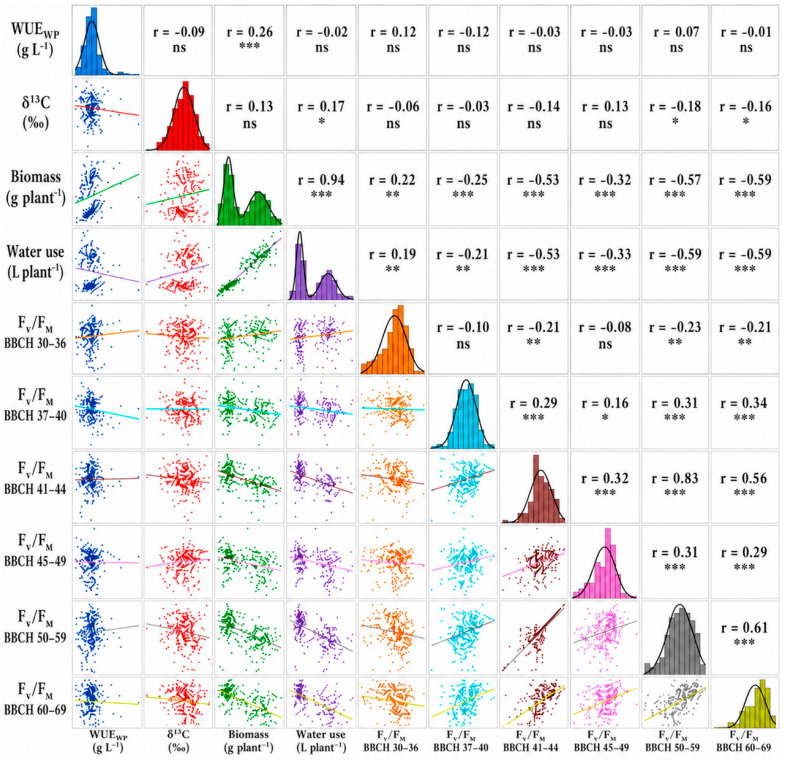
Variation and Spearman scatter plots correlations among traits. Histograms for WUE_WP_, δ^13^C, biomass, and water use per plant values measured are displayed along the diagonal. The frequency distribution of each variable is shown on the diagonal. On the bottom of the diagonal, the bivariate scatter plots with a fitted line are displayed. On the top of the diagonal: the values of the correlations plus the significance level as ‘ns’, *, ** and *** are significance levels of 0.1, 0.05, 0.001 (not-significant, significant, very significant, and highly significant), respectively. The experiments were conducted in a BioChamber’s plant growth room, Conviron BioChamber LTRB Growth Room, at the University of Alberta in Edmonton, Alberta. The values were measured on the whole plant in two replicates and four samplings per plant. The regression lines are presented for visualization purposes only and all reported correlation coefficients and significance tests are based on Spearman’s rank correlation analysis.

The 198 wheat cultivars were evaluated in two growth-chamber runs, each comprising 99 unique cultivars, due to space limitations. Both runs were conducted under the same environmental conditions; however, because no common check cultivars were included across runs, cultivar comparisons are valid only within each run, and differences between runs should be interpreted cautiously. Significant variation was observed among cultivars for WUE_WP_, biomass, δ^13^C, and water use (Table 1). Biomass and water use exhibited bimodal distributions, indicating two contrasting groups of cultivars with different growth and water-consumption patterns, although some influence of run-to-run variation cannot be excluded. In contrast, WUE_WP_ and δ^13^C showed unimodal distributions with lower variability, suggesting more consistent physiological regulation across the cultivar panel (Figure 3).

Spearman correlation analysis revealed substantial phenotypic diversity among cultivars (Figure 3). Biomass was strongly positively correlated with water use (r = 0.94, *p* < 0.001), indicating that greater biomass production was associated with higher water consumption. In contrast, WUE_WP_ was only weakly related to biomass (r = 0.26, *p* < 0.001), δ^13^C (r = −0.09), and water use (r = −0.02), suggesting that whole-plant water-use efficiency is governed by multiple interacting physiological processes.

Relationships between F_V_/F_M_ and agronomic traits varied across developmental stages under non-stress (BBCH 30–36, 37–40, 50–59, and 60–69) and combined drought–heat stress conditions (BBCH 41–44 and 45–49). F_V_/F_M_ showed consistently weak correlations with WUE_WP_ and δ^13^C (r = −0.18 to 0.13), indicating limited direct relationships between PSII photochemical efficiency and long-term water-use efficiency metrics. In contrast, F_V_/F_M_ was negatively correlated with biomass (r = −0.53 to −0.59, *p* < 0.001) and water use (r = −0.32 to −0.59, *p* < 0.001), with the strongest associations observed after the stress period (BBCH 50–69). These findings indicate that PSII photochemical efficiency was more closely linked to plant growth and water use than to WUE_WP_ or δ^13^C, supporting F_V_/F_M_ as a sensitive indicator of wheat responses to combined drought and heat stress.

### 2.3. Hierarchical Clustering and Heatmap Analysis of Physiological Traits

**Figure 4:**
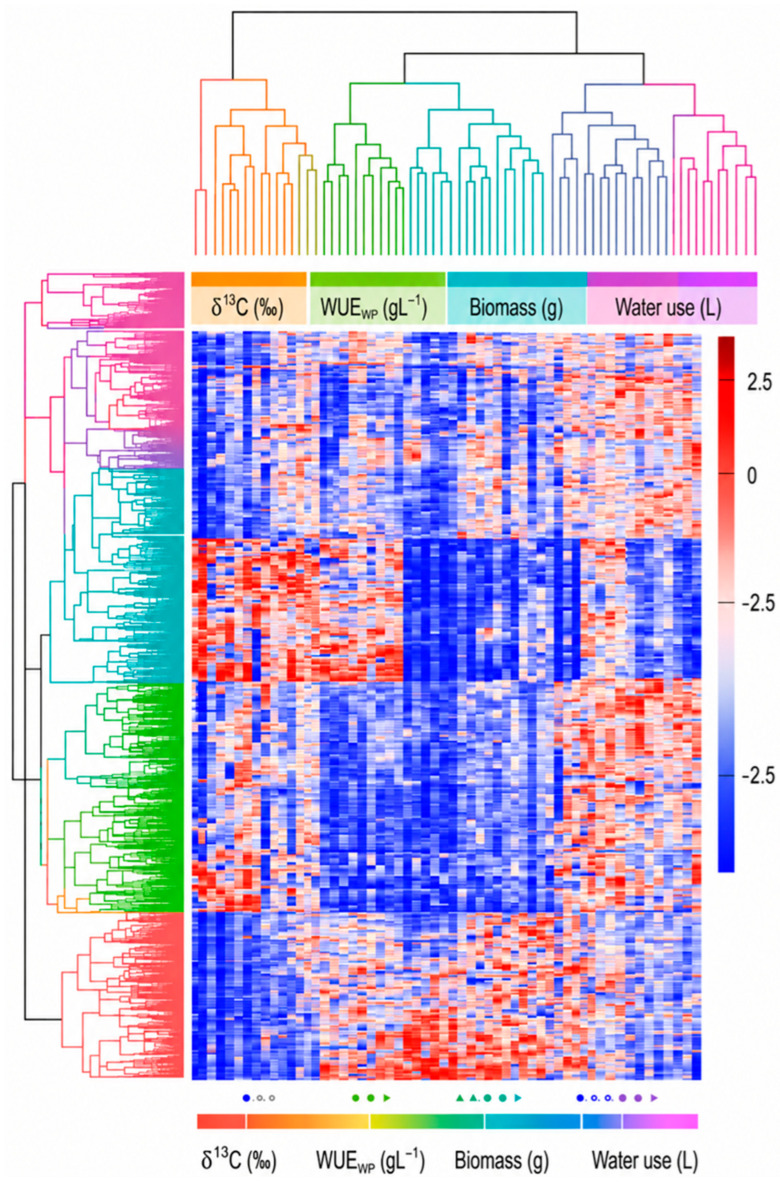
Hierarchical clustering heatmap of genotypes based on δ^13^C (‰), WUE_WP_ (g L^−1^), biomass (g plant^−1^), and water use (L plant^−1^). Rows (genotypes) and columns (traits) are clustered using hierarchical clustering (dendrograms shown on the left and top). Values are standardized (z-scores). Color scale indicates relative trait values: red = higher, blue = lower, and white = near the mean (approximately −2.5 to +2.5). Distinct clusters highlight contrasting trait combinations, particularly separating high-biomass/high-water-use genotypes from lower productivity groups with variable efficiency.

Hierarchical clustering and heatmap analyses revealed substantial phenotypic variation among the 198 Canadian spring wheat cultivars for δ^13^C, WUE_WP_, biomass accumulation, and water use per plant under combined drought and heat stress conditions (Figure 4). The hierarchical dendrogram separated the cultivar panel into several major clusters and sub-clusters, reflecting diverse physiological responses associated with carbon assimilation, water acquisition, and biomass production. Red and blue heatmap gradients represented standardized trait values above and below the population mean, respectively, highlighting distinct physiological profiles among cultivars. Cluster assignments are provided in Supplementary Table S1.

Biomass accumulation and water use exhibited similar clustering patterns, consistent with the strong positive association observed between these traits. In general, cultivars with greater biomass accumulation clustered with higher water use, whereas cultivars with lower biomass tended to exhibit reduced water consumption. However, several cultivars deviated from this trend, indicating differences in water-use efficiency. Several cultivars distinguished themselves by combining above-average biomass accumulation with below-average water use, including AAC Iceberg (26.22 g plant^−1^; 5.30 L plant^−1^), AAC Tradition (33.61 g plant^−1^; 6.11 L plant^−1^), Kenyon (33.07 g plant^−1^; 5.13 L plant^−1^), and Pasqua (27.93 g plant^−1^; 5.89 L plant^−1^), representing approximately 2.0% of the evaluated germplasm. In contrast, 89 cultivars (44.9%) exhibited both above-average biomass and water use, whereas only five cultivars (2.5%)—CDC Terrain (24.02 g plant^−1^; 7.50 L plant^−1^), Harvest (23.72 g plant^−1^; 6.80 L plant^−1^), Invader (25.07 g plant^−1^; 7.40 L plant^−1^), Minnedosa (25.17 g plant^−1^; 8.32 L plant^−1^), and Parata (24.68 g plant^−1^; 7.20 L plant^−1^)—combined below-average biomass with above-average water use. These contrasting phenotypes highlight substantial variation in biomass production per unit of water consumed and identify promising germplasm for improving drought adaptation.

Distinct clustering patterns were also evident for δ^13^C; however, δ^13^C did not consistently co-cluster with WUE_WP_ across genotype groups. Cultivars with similar δ^13^C values frequently exhibited contrasting WUE_WP_ responses, suggesting that carbon isotope composition alone does not fully explain variation in whole-plant water-use efficiency under combined drought and heat stress. This pattern indicates that WUE_WP_ is regulated by multiple interacting physiological processes, including photosynthetic carbon assimilation, stomatal conductance, transpiration, and biomass production.

Overall, the clustering analysis revealed diverse physiological strategies for coping with drought and heat stress within Canadian spring wheat germplasm. The identification of cultivars capable of sustaining high biomass accumulation with relatively low water consumption demonstrates the presence of exploitable phenotypic variation for improving water-use efficiency and stress adaptation. However, the limited number of cultivars exhibiting this favorable trait combination suggests that incorporating more diverse germplasm may be necessary to achieve substantial future gains in climate resilience and water-use efficiency.

### 2.4. Variation in Chlorophyll Fluorescence Parameters

**Figure 5:**
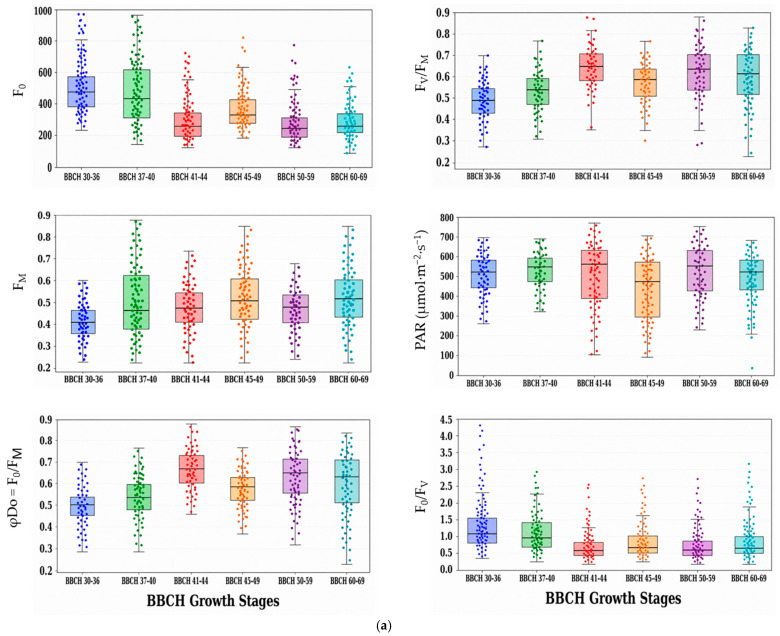

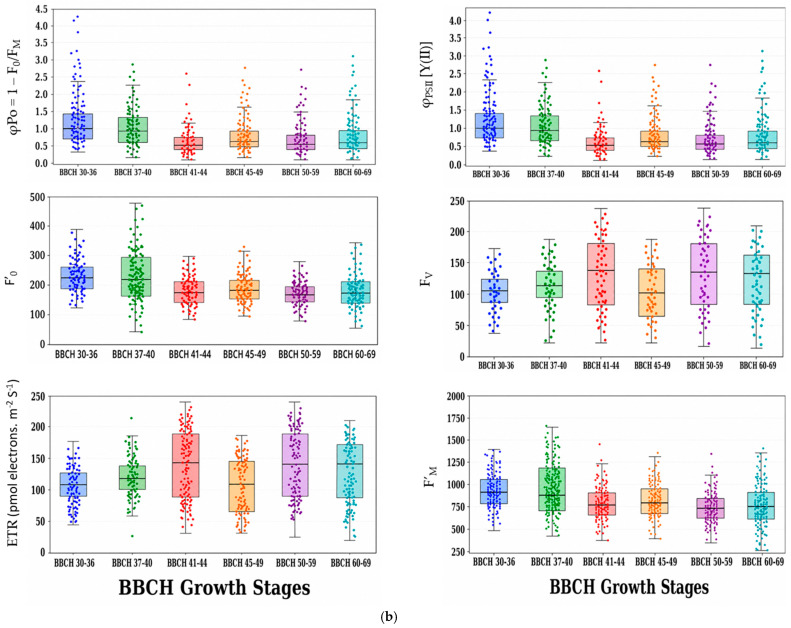
(**a**). Boxplot distributions of chlorophyll fluorescence parameters including F_0_, F_V_/F_M_, F_M_, PAR, φDo, and F_0_/F_V_ measured in 198 Canadian spring wheat cultivars across six BBCH growth stages under non-stress and stress conditions. (**b**). Boxplot distributions of chlorophyll fluorescence parameters, including φPSII (YII), F′_0_, F_V_, ETR, F′_M_, and φPo measured in 198 Canadian spring wheat cultivars across six BBCH growth stages, with BBCH 45–49 representing seven days of combined drought and heat stress.

Considerable variation in chlorophyll fluorescence parameters was observed among the 198 Canadian spring wheat cultivars across the six BBCH growth stages evaluated under both non-stress and stress conditions (Figure 5a,b). Variations in median values, interquartile ranges, and overall distribution patterns demonstrated substantial genotypic diversity in photosynthetic performance and physiological responses to drought and heat stress throughout plant development.

The chlorophyll fluorescence parameters could generally be grouped into three major physiological response patterns, particularly during the stress-associated reproductive stage BBCH 45–49, corresponding to seven days of combined drought and heat stress. The first group, comprising F_V_/F_M_, φDo, F_V_, and ETR, exhibited comparatively greater variability and broader interquartile ranges during BBCH 45–49. These parameters showed substantial dispersion among cultivars and the presence of several outliers, indicating substantial genotypic differences in PSII efficiency, photochemical activity, and electron transport capacity under stress conditions. Several cultivars maintained relatively higher values during BBCH 45–49, suggesting improved stability of photosynthetic processes and enhanced tolerance to combined drought and heat stress. The second group included F_0_, F_0_/F_V_, φPSII (YII), F′_0_, and F′_M_, which generally displayed reductions during BBCH 45–49 relative to the earlier non-stress stages BBCH 30–36 and BBCH 37–40. The decline in these parameters suggests impairment of PSII stability and alterations in excitation energy utilization under stress conditions. In addition, the wider distribution patterns observed during BBCH 45–49 indicate differential cultivar responses in energy dissipation and photochemical regulation mechanisms. Nevertheless, several cultivars maintained comparatively stable parameter values, reflecting potential adaptive physiological mechanisms associated with stress tolerance. The third group consisted of PAR and F_M_, which exhibited comparatively moderate variation during BBCH 45–49 relative to the other fluorescence traits. Although fluctuations among cultivars were observed, these parameters remained relatively more stable during the stress treatment, suggesting lower sensitivity to stress progression compared with PSII efficiency-related traits.

Overall, BBCH 45–49 exhibited some of the greatest variability in chlorophyll fluorescence responses among all evaluated growth stages. The pronounced variation observed across fluorescence parameters during this reproductive stress stage highlights substantial genotypic diversity in the ability of Canadian spring wheat cultivars to maintain PSII stability and photosynthetic efficiency under combined drought and heat stress conditions. These findings indicate the presence of exploitable physiological variation for improving stress resilience and photosynthetic stability in future wheat breeding programs.

### 2.5. Principal Component Analysis (PCA) Among Agronomic and Water-Related Traits

**Figure 6:**
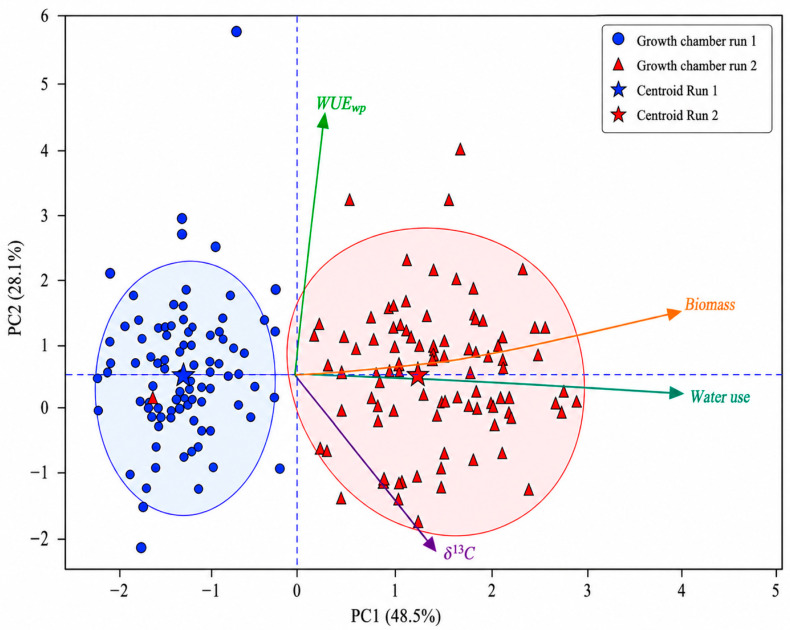
PCA analysis of 198 Canadian spring wheat cultivars derived from various breeding programs. The *x*-axis and *y*-axis represent principal component 1 (PC 1) and principal component 2 (PC 2), respectively, with the proportions. Presented here are the following: WUE_WP_, δ^13^C, biomass and water use per plant.

Principal component analysis (PCA) revealed clear separation among the 198 Canadian spring wheat cultivars based on WUE_WP_, δ^13^C, biomass accumulation, and water use per plant (Figure 6). The first two principal components explained 76.6% of the total variation, with PC1 and PC2 accounting for 48.5% and 28.1%, respectively. Biomass accumulation and water use were strongly associated with the positive axis of PC1, indicating a close positive relationship between plant growth and water consumption. In contrast, WUE_WP_ and δ^13^C were more strongly associated with PC2, suggesting distinct physiological relationships with biomass accumulation and water use. The separation of WUE_WP_ from biomass and water use indicates that increased WUE_WP_ was not consistently associated with greater biomass production or water consumption across cultivars. Similarly, the distinct orientation of the δ^13^C vector suggests variation in carbon assimilation and stomatal regulation among genotypes.

The PCA biplot further revealed two major cultivar groups primarily distributed along PC1, which may partly reflect differences in growth performance and potential variation between growth-chamber runs. Cultivars grouped on the positive side of PC1 were generally associated with greater biomass accumulation and water use, whereas cultivars on the negative side were associated with lower growth and reduced water consumption. In contrast, WUE_WP_ and δ^13^C appeared less influenced by these factors, as their vector orientations were not strongly associated with the separation between the two major cultivar groups.

Overall, the broad dispersion of cultivars across the PCA space highlights substantial physiological diversity and the presence of exploitable variation for improving WUE_WP_, drought adaptation, and stress resilience in Canadian spring wheat breeding programs. However, despite the observed variability, the overall magnitude of phenotypic divergence among cultivars appeared relatively limited, suggesting that the existing diversity within the current Canadian spring wheat germplasm may not be sufficient to support major genetic gains for WUE improvement through conventional breeding alone.

## 3. Discussion

### 3.1. Phenotypic and Physiological Variation in Canadian Spring Wheat Cultivars

The inclusion of historical and modern Canadian spring wheat cultivars spanning more than a century of breeding enabled a comprehensive assessment of variation in WUE_WP_, δ^13^C, biomass accumulation, water use, and chlorophyll fluorescence under combined drought and heat stress. Correlation analysis, principal component analysis, hierarchical clustering, and the Shannon diversity index were used to evaluate physiological diversity, photosynthetic performance, and stress adaptation. Substantial variation in biomass, water use, WUE_WP_, and δ^13^C indicated considerable physiological diversity among cultivars. However, the narrower distributions of WUE_WP_ and δ^13^C compared with biomass and water use suggest that these traits are relatively conserved and influenced by both genetic and environmental factors. Cultivars with higher WUE_WP_ likely achieve greater carbon assimilation and biomass production per unit of water consumed under stress.

Previous studies similarly reported substantial variation in wheat WUE across environments, developmental stages, and management conditions, emphasizing the strong influence of genotype × environment interactions on WUE [49,50,51,52]. The relatively stable range of δ^13^C observed among cultivars further suggests that δ^13^C may reflect long-term physiological regulation of stomatal conductance and photosynthetic activity under stress conditions. Overall, these findings demonstrate that exploitable variation exists for WUE-related traits within Canadian spring wheat germplasm and highlight the potential for integrating physiological traits such as WUE_WP_ and δ^13^C into breeding programs aimed at improving WUE_WP_, drought adaptation, and climate resilience. However, the magnitude of the observed diversity may not be sufficiently broad to support rapid genetic gains, suggesting that historical breeding has already narrowed the genetic base for these adaptive traits.

### 3.2. Phenotypic Diversity and Breeding Program Structure

The variation observed among breeding programs for WUE_WP_, δ^13^C, water use, and biomass demonstrated moderate to substantial phenotypic diversity within Canadian spring wheat germplasm, although the magnitude of variation was influenced in part by the number of cultivars represented within each program. Distinct trait distributions indicate that breeding history, parental selection, germplasm sources, target environments, and regional adaptation have shaped contrasting physiological responses to drought and heat stress. Programs exhibiting broader trait ranges likely possess wider genetic backgrounds and greater opportunities for selecting stress-adaptive traits, whereas narrower distributions may reflect stronger directional selection and reduced phenotypic diversity [53,54,55].

Differences among breeding programs also reflect contrasting historical breeding objectives, including selection for yield potential, disease resistance, end-use quality, and adaptation to Prairie environments. Programs utilizing a broader range of parental lines generally retained greater variation in physiological traits such as WUE_WP_, δ^13^C, biomass accumulation, and water use per plant. Although the AAFC breeding programs contributed the largest number of cultivars to the panel, they exhibited a relatively narrow range of variation for WUE_WP_, suggesting shared genetic backgrounds or similar historical selection pressures. Similar trends have been reported in modern wheat breeding programs, where recurrent use of elite parental lines and selection bottlenecks can reduce phenotypic and physiological diversity unless novel germplasm is introduced [56,57,58].

The Shannon diversity index values in the wheat panel ranged from 1.43 to 2.62, indicating low to medium diversity, where values near 1 represent low diversity and values around 2.5 indicate moderate diversity. Higher Shannon diversity values reflect greater trait diversity [59,60], indicating that the evaluated spring wheat cultivars retain useful phenotypic variation for improving WUE_WP_, drought tolerance, and biomass production. Overall, these results suggest a moderate level of diversity for traits related to WUE_WP_, δ^13^C, biomass accumulation, and water use per plant, consistent with previous reports in wheat [61,62]. Although the observed diversity may be adequate for some specific breeding objectives, substantial improvement in WUE_WP_ and associated physiological traits would likely require the incorporation of additional phenotypic variation. Strategic introduction of diverse international germplasm could broaden the adaptive potential of Canadian spring wheat under increasingly variable climatic conditions. The continuing erosion of phenotypic diversity among modern, high-yielding varieties remains a concern, highlighting the need for coordinated regional and global efforts to broaden the wheat genetic base and support long-term crop resilience [63,64,65,66,67,68].

### 3.3. Carbon Isotope Discrimination and Water-Use Efficiency Relationships

The broad range observed (24.06–29.33‰) indicated substantial genotypic variation and suggested differences in stomatal regulation, mesophyll conductance, and photosynthetic capacity among cultivars. Such variation suggested that the plants exhibited differential physiological responses to drought and heat stress, reflecting differences in stomatal regulation, mesophyll conductance, and photosynthetic capacity. Plants with low δ^13^C values tend to exhibit conservative water-use behavior under heat stress, characterized by reduced stomatal conductance and enhanced WUE, whereas those with higher δ^13^C values tend to favor a more acquisitive strategy to sustain higher carbon assimilation rates despite greater water loss. These physiological trade-offs highlight the complexity of breeding for drought and heat tolerance, as the optimal balance between productivity and water conservation depends on the target environment and breeding objectives [69,70].

The negative relationship between WUE_WP_ and δ^13^C, commonly reported in previous studies, may be due to physiological mechanisms that regulate δ^13^C during photosynthesis. During CO_2_ fixation, plants preferentially assimilate the lighter carbon isotope (^12^C) over the heavier isotope (^13^C). When stomatal conductance is high and intercellular CO_2_ concentration (Ci) approaches ambient levels, discrimination against ^13^C increases, leading to higher δ^13^C values. In contrast, when stomatal conductance is reduced under stress conditions, Ci declines, leading to lower discrimination and more negative δ^13^C values. As a result, lower δ^13^C values are generally associated with higher intrinsic WUE, reflecting a balance between maintaining photosynthetic carbon assimilation and minimizing water loss through transpiration [71,72].

### 3.4. Phylogenetic and Market Class Analysis of Physiological Traits

The hierarchical clustering dendrogram illustrating variation in WUE_WP_, δ^13^C, biomass accumulation, and water use per plant among Canadian spring wheat cultivars revealed distinct groupings of cultivars with similar physiological profiles, suggesting that genetic background may contribute to the expression of these traits. The clustering patterns demonstrated substantial phenotypic variation in water-use strategies, biomass production, and carbon assimilation under drought and heat stress, highlighting the complex interactions among physiological mechanisms governing stress adaptation. The association between δ^13^C and WUE_WP_ further supports the use of δ^13^C as an indirect indicator of WUE. Similar variability in WUE and stress-adaptive traits has been reported in wheat, emphasizing the importance of physiological diversity for breeding climate-resilient cultivars [73,74]. The broad diversity observed among cultivars originating from different Canadian breeding programs suggests the development of multiple adaptive response strategies to environmental stress. Nevertheless, the persistence of considerable variation among cultivars indicates substantial opportunities for further improvement of drought and heat resilience through physiological trait-based selection.

A strong clustering association was observed between biomass accumulation and water use per plant, indicating that increased productivity was generally associated with greater water consumption. This relationship is consistent with previous findings showing that higher biomass production in wheat is often linked to increased transpiration and water uptake due to enhanced photosynthetic activity and canopy development [75,76]. Although greater water use may support higher growth potential, it may also increase vulnerability under prolonged drought conditions, particularly in water-limited environments. Consequently, balancing biomass production with efficient water utilization remains a major challenge in wheat improvement programs.

The observed variation in WUE among cultivar clusters further demonstrated that some genotypes maintained relatively high biomass production despite moderate water use, indicating improved physiological efficiency under stress conditions. These cultivars may possess adaptive mechanisms such as improved stomatal regulation, enhanced osmotic adjustment, or more efficient carbon assimilation processes that contribute to superior WUE performance. Recent studies have similarly reported that genotypic differences in WUE are controlled by complex interactions among transpiration efficiency, photosynthetic capacity, and stress-responsive physiological traits [77,78].

The inconsistent clustering relationship between δ^13^C and WUE suggests that carbon isotope discrimination alone may not adequately predict whole-plant WUE under combined drought and heat stress conditions. Although δ^13^C has frequently been used as an indirect indicator of transpiration efficiency and stomatal behavior, its relationship with WUE can vary depending on environmental conditions, developmental stage, and genotype-specific physiological responses [79,80]. The weak co-clustering observed in the present study indicates that WUE in wheat is influenced by multiple physiological processes beyond carbon isotope discrimination alone, including biomass partitioning, transpiration dynamics, and stress-induced metabolic adjustments.

Canadian wheat cultivars were grouped according to official marketing classes defined primarily by grain quality and end-use characteristics. We hypothesized that WUE_WP_, δ^13^C, biomass accumulation, and water use would differ among classes due to underlying physiological variation; however, no significant differences were detected for any trait. This suggests that Canadian wheat classes share broadly similar physiological responses despite differences in grain quality, likely reflecting a common breeding history focused on grain yield, end-use quality, and disease resistance rather than water-use efficiency traits. Nevertheless, the substantial variation observed among individual cultivars indicates the presence of exploitable phenotypic diversity for improving WUE_WP_, drought adaptation, and stress resilience. These findings highlight opportunities to incorporate physiological traits such as WUE_WP_, biomass accumulation, chlorophyll fluorescence, and δ^13^C into breeding programs to accelerate the development of climate-resilient wheat cultivars adapted to increasingly variable environmental conditions.

### 3.5. Chlorophyll Fluorescence Responses and PSII Stability Under Combined Drought and Heat Stress

Chlorophyll fluorescence, particularly the maximum quantum efficiency of photosystem II F_V_/F_M_, is a sensitive indicator of drought and heat stress in wheat and is closely associated with photosynthetic efficiency, WUE, and stress adaptation. F_V_/F_M_ provides a rapid and non-destructive assessment of stress-induced damage to PSII [81,82,83,84,85]. In this study, substantial variation in F_V_/F_M_ among the 198 Canadian spring wheat cultivars revealed considerable diversity in photosynthetic resilience under both combined drought and heat stress, with ‘CDC Teal’ maintaining the highest values and ‘Superb’ the lowest.

F_V_/F_M_ measured during non-stress and recovery stages (BBCH 30–36, 37–40, 50–59, and 60–69) showed relatively weak relationships with biomass accumulation and water use, indicating stable photosynthetic activity under favorable conditions. In contrast, F_V_/F_M_ during reproductive stress stages (BBCH 41–44 and 45–49) exhibited stronger negative relationships with biomass and water use, indicating that combined drought and heat stress impaired PSII efficiency, electron transport, and plant productivity. Greater variation in F_V_/F_M_, φDo, FV, and ETR during BBCH 45–49 further confirmed strong stress effects on PSII photochemistry and electron transport processes. Similar responses have been reported in tolerant cereal genotypes maintaining higher chlorophyll fluorescence efficiency under drought and heat stress [86,87,88,89,90].

Temporal changes in fluorescence parameters reflected dynamic physiological adjustments to stress. Short-term increases in PAR, ETR, and F_V_/F_M_ from stem elongation to booting suggested transient acclimation responses associated with photoprotective mechanisms [91,92]. However, prolonged stress reduced these parameters, indicating PSII impairment, reduced carbon assimilation, and photo-oxidative damage [93]. Cultivars maintaining stable fluorescence responses under stress likely possess adaptive physiological mechanisms contributing to improved heat tolerance and WUE_WP_. Reductions in F_V_/F_M_ were closely associated with decreases in biomass accumulation and potential productivity, reflecting photoinhibition and structural damage to PSII that constrain electron transport and carbon assimilation [5,81,94,95]. Combined drought and heat stress can cause substantial yield losses in wheat [6,96,97]. Genotypes maintaining higher F_V_/F_M_ values under stress exhibited greater PSII stability, sustained photosynthesis, and improved biomass production, highlighting the importance of chlorophyll fluorescence traits for identifying drought- and heat-tolerant germplasm [1,95,98,99].

PCA analysis further revealed substantial physiological diversity among cultivars for WUE_WP_, δ^13^C, biomass accumulation, and water use under climate stress. The first two principal components explained 76.6% of the total variation, indicating that a limited number of physiological traits accounted for most cultivar variability [1,63]. Biomass accumulation and water use were strongly associated with PC1, whereas WUE_WP_ and δ^13^C were more closely related to PC2, representing a distinct physiological axis associated with water-use efficiency and carbon isotope discrimination. These results highlight the complex interaction among WUE_WP_, heat tolerance, photosynthetic efficiency, and δ^13^C and emphasize the importance of integrating these physiological traits into breeding programs aimed at improving climate resilience in Canadian spring wheat.

## 4. Materials and Methods

### 4.1. Plant Material

A spring wheat diversity panel consisting of 198 historical and modern Canadian cultivars registered in western Canada between 1905 and 2018 was used in this study (Table 2). These cultivars represent releases from more than eleven breeding programs across Canada. The panel encompasses a wide range of genetic backgrounds and end-use quality types, capturing the historical and contemporary phenotypic diversity of Canadian spring wheat. Cultivars were classified according to their official marketing classes, which are defined by functional characteristics such as grain color, hardness, kernel size, baking and milling quality, dough or gluten strength, grain protein concentration, and end-use [100]. The classes are Canada Northern Hard Red (CNHR), Canada Prairie Spring Red (CPSR), Canada Prairie Spring White (CPSW), Canada Western Amber Durum (CWAD), Canada Western Extra Strong (CWES), Canada Western Hard White Spring (CWHWS), Canada Western Red Spring (CWRS), Canada Western Special Purpose (CWSP), and Canada Western Soft White Spring (CWSWS) (Supplementary Table S2). Among these, the CWRS class is the most widely cultivated, accounting for approximately 60% of Canada’s total wheat production, followed by CWAD, CPSR, and CESRW [101]. The inclusion of cultivars from these classes ensured comprehensive representation of phenotypic variation in agronomic and physiological traits related to water-use efficiency, drought tolerance, and heat stress response.

### 4.2. Controlled Growth Conditions and Drought–Heat Stress Treatment

Plants were grown in a controlled-environment Conviron BioChambers LTRB growth room equipped with programmable LED lighting and automated environmental control systems located at the University of Alberta, Edmonton, Canada. The chamber conditions were maintained under an 18 h light/6 h dark photoperiod with a photosynthetic photon flux density (PPFD) of approximately 1000 µmol m^−2^ s^−1^ measured at canopy level to support optimal photosynthetic activity and plant development. Day/night air temperatures were maintained at 22/16 °C, while relative humidity was regulated at approximately 55% during the light period and 70% during the dark period to minimize excessive transpiration and maintain stable plant water status. The growth room was equipped with forced-air circulation and ventilation systems to ensure uniform distribution of temperature, humidity, and CO_2_ throughout the chamber and to reduce microenvironmental variation among pots. Environmental parameters, including temperature, relative humidity, light intensity, and photoperiod, were continuously monitored and digitally controlled using the Conviron environmental control system. The LED lighting system provided a broad-spectrum light source designed to simulate natural daylight conditions and support uniform plant growth across developmental stages.

Individual plants were grown in 1-gallon pots (≈3.79 L) containing a soil mixture composed of field soil and peat moss (Promix BX) at a 1:3 (*v*/*v*) ratio. Pots were arranged in a completely randomized design and periodically repositioned within the growth chamber to minimize positional effects. Prior to sowing, all pots were saturated with tap water and allowed to drain overnight to determine field capacity. Three seeds were sown per pot at a depth of 1.5 cm, and seedlings were thinned to one plant per pot two weeks after emergence. To minimize soil evaporation, the soil surface was covered with a 2 cm layer of perlite. Plants were maintained at field capacity through daily gravimetric watering based on pot weight until the initiation of stress treatments. During the experiment, plants were fertilized once weekly with a liquid fertilizer applied through irrigation at the manufacturer’s recommended rate to maintain adequate nutrient availability and support uniform growth across all plants.

Each genotype was evaluated under water-deficit and heat stress conditions using two biological replicates and four measurements per plant in a controlled experiment. Combined drought and heat stress treatments were imposed at the booting stage (BBCH 41–44) and BBCH (45–49) according to the Zadoks growth scale [102], a critical reproductive stage highly sensitive to environmental stress in wheat. Heat stress was applied by increasing the temperature from 22 °C to 35 °C, and the elevated temperature was maintained for seven consecutive days under water-deficit conditions to simulate combined climate stress.

Drought Stress Treatment. Drought stress was imposed during the reproductive stage by withholding irrigation until the target soil water content (SWC) was reached. Prior to stress imposition, pots were maintained under well-watered conditions at approximately 70–100% of field capacity. Soil moisture was then allowed to decline gradually until SWC reached approximately 10–15%, after which plants were maintained under a controlled water-deficit regime for 7 days. Throughout the stress period, soil moisture was maintained near 10% SWC through daily gravimetric replacement of water lost via evapotranspiration, ensuring a uniform and severe drought treatment across all cultivars.

Soil moisture was monitored independently using a 20 mm stainless-steel three-rod soil moisture probe (Soil Moisture Equipment Corp., Santa Barbara, CA, USA) and verified through daily pot weighing. Each pot was weighed at the same time each day, and the amount of water required to maintain the target SWC was calculated gravimetrically. This approach minimized variation in soil moisture among pots and ensured consistent stress intensity throughout the experiment.

To improve the accuracy of water-use measurements, two unplanted control pots containing the same soil substrate were maintained under identical environmental conditions and weighed daily to quantify evaporative water loss. Plant water use was subsequently calculated after correcting for evaporation from the substrate surface. The gravimetric method provided precise control of soil moisture and ensured comparable drought stress among cultivars despite differences in plant size, growth rate, and transpiration demand.

At the end of the 7-day drought treatment, corresponding to an average soil water potential of approximately −80 kPa, all pots were rewatered to field capacity and maintained under well-watered conditions until physiological maturity. Plants were then harvested for the determination of biomass accumulation, water-use efficiency, carbon isotope composition (δ^13^C), and other physiological measurements.

Soil water content was calculated as:

(1) $$SWC(\%)=\frac{W_{actual}-W_{min}}{W_{max}-W_{min}}\times 100$$

where $W_{actual}$ is the measured pot weight on a given day, $W_{min}$ is the dry pot weight, and $W_{max}$ is the field-capacity pot weight.

### 4.3. Determination of WUE at the Whole Plant Level

Whole-plant water consumption throughout the growth period until physiological maturity was estimated gravimetrically from the cumulative daily water loss determined by pot weight measurements. Daily water consumption was calculated as the difference between pot weight at field capacity and the corresponding daily pot weight, as follows:

(2) $$\mathrm{Whole}-\mathrm{Plant}\;\mathrm{Water}\;\mathrm{Consumption}\;=\;\left(\mathrm{Pot}\;\mathrm{Weight}\;\mathrm{at}\;\mathrm{Field}\;\mathrm{Capacity}\right)-\left(\mathrm{Daily}\;\mathrm{Pot}\;\mathrm{Weight}\right)$$

At the plant maturity stage (BBCH 83), WUE was estimated as the ratio of total above-ground biomass accumulation to total water applied during the experiment, less water lost due to evaporation. WUE was determined as follows:

(3) $$WUE_{WP}\;\left(g\;L^{-1}\right)=\frac{\mathrm{Dry}\;\mathrm{weight}\;\mathrm{of}\;\mathrm{final}\;\mathrm{biomass}\;(g)}{\mathrm{Total}\;\mathrm{water}\;\mathrm{consumed}\;(L)}$$

where $WUE_{WP}$ = whole-plant water-use efficiency

Dry weight of final biomass = total dry biomass produced per plant (g)
Total water consumed = cumulative water used per plant during the experiment (L)

### 4.4. Determination of Carbon Isotope Discrimination (δ^13^C) in Flag Leaf

For δ^13^C analysis, all flag leaves (5–10 leaves per plant, depending on tiller number) from plants subjected to combined drought and heat stress were harvested and bulked for each plant to obtain a representative sample for isotopic determination. Leaf samples were air-dried, finely ground, and subsamples of approximately 2 mg were submitted to the Analytical Service Laboratory, Faculty of Agricultural, Life and Environmental Sciences—Renewable Resources, University of Alberta, for analysis. Carbon isotope composition (δ^13^C relative to VPDB) was determined by flash combustion coupled with continuous-flow isotope ratio mass spectrometry (CF-IRMS). During analysis, samples were combusted in oxygen to convert carbon into CO_2_, which was subsequently separated chromatographically and analyzed by IRMS. The naturally occurring stable carbon isotopes consist primarily of ^12^C (98.89%) and ^13^C (1.11%). Working standards were calibrated against the international VPDB reference standard, and raw isotope data were normalized to VPDB values using linear regression derived from standard measurements. Analyses were performed using a Thermo Delta V Advantage Isotope Ratio Mass Spectrometer (Thermo Scientific Inc., Bremen, Germany) coupled with a Thermo FLASH HT Plus 2000 Organic Elemental Analyzer and ConFlo IV (Thermo Fisher Scientific Inc., Bremen, Germany) for continuous-flow isotope ratio mass spectrometry (CF-IRMS) (CF-IRMS).

(4) $$\delta^{13}C_{\mathrm{sample}}\;(‰)=\frac{R_{sample}-R_{std}}{R_{std}}$$

where δ^13^C_sample_ is that of the sample of interest, R_sample_ is its ^13^C/^12^C ratio, and R_std_ is the ^13^C/^12^C ratio of a standard. The δ^13^C values were referenced to a Pee Dee Belemnite standard, which is the internationally accepted standard for expressing stable carbon isotope ratios, with a ^13^C/^12^C of 0.0112372 [103]. In order to avoid working with very small numbers, Δ and δ^13^Csample are typically multiplied by 1000 and expressed in parts per thousand (‰).

### 4.5. Chlorophyll Fluorescence Measurements and Physiological Assessment Under Drought–Heat Stress

For the determination of chlorophyll a fluorescence yield, readings were taken with a pulse fluorometer of modulated amplitude (OS5-FL Modulated Chlorophyll Fluorometer, OPTI-SCIENCES), using leaves submitted to ambient light (when the photosynthetic apparatus is at full capacity) and leaves submitted to darkness (when the photosynthetic apparatus is fully recovered) according to Schreiber et al. [104]. The optic fiber was maintained at a constant distance of 2 cm at an angle of approximately 60° in relation to the adaxial surface of the leaf. The light pulses had a duration of 0.8 s and an intensity of 1000 μmol m^−2^ s^−1^. In leaves submitted to ambient light, readings were taken between 9:00 and 11:00 a.m. (with the same leaves used for fluorescence emission at dawn). The following chlorophyll *a* fluorescence parameters were calculated: maximum quantum efficiency of PSII primary photochemistry (F_V_/F_M_), according to van Kooten and Snel [105]; effective quantum yield of PSII [ΦPSII = (F′_M_ − FS)/F′_M_], according to Genty et al. [106]; apparent rate of photochemical transport of electrons through PSII (ETR = ΦPSII × PAR × 0.5 × 0.84), according to Schultz [107]. The coefficient of photochemical quenching [qL = ((F′_M_ − FS)/F′_M_) − F′_0_ × F′_0_/FS] was determined according to Kramer et al. [108] and the coefficient of non-photochemical quenching of excitation energy [NPQ = (F_M_ − F′_M_)/F′_M_] according to Schreiber et al. [104]. Where F0 = initial fluorescence in leaves submitted to a period of darkness; F_M_ = maximum fluorescence in leaves acclimated to darkness; F_V_ = variable fluorescence in leaves acclimated to darkness (F_V_ = F_M_ − F_0_); F′_0_ = initial fluorescence in leaves submitted to ambient light; F′_M_ = maximum fluorescence in leaves acclimated to ambient light; FS = stable fluorescence in leaves acclimated to ambient light; PAR = incident photosynthetically active radiation in the leaf; 0.5 corresponds to the proportion of absorbed quanta that are used by the PSII reaction centers; and 0.84 represents the proportion of incident irradiance that is absorbed by the leaf.

Chlorophyll fluorescence parameters were measured before stress imposition, during the stress period, and following recovery to evaluate the physiological responses of wheat cultivars to combined drought and heat stress. Measurements were conducted at the center of the flag leaf of each tiller, as the flag leaf is considered the primary photosynthetically active organ during grain filling and contributes approximately 45–58% of total photosynthetic activity in wheat [109]. Four technical measurements were recorded per plant across the two biological replicates, resulting in a total of eight observations per genotype for each parameter.

### 4.6. Experimental Design and Data Analysis

The experiment was arranged in a completely randomized design with two biological replicates, where each plant served as an experimental unit. Two control pots containing the same soil mixture and perlite, but no plants, were included to estimate soil surface evaporation. For chlorophyll fluorescence measurements, four technical readings were collected per plant at each growth stage, resulting in eight observations per fluorescence parameter across biological replicates.

Statistical analyses were conducted using SAS version 9.4 (SAS Institute Inc., Cary, NC, USA). Chlorophyll fluorescence (F_V_/F_M_) data were analyzed using repeated-measures analysis of variance (repeated-measures ANOVA) implemented in PROC MIXED, with cultivar treated as the subject and growth stage (BBCH stage) specified as the repeated factor. Fixed effects included cultivar, growth stage, and their interaction, while repeated observations within cultivars were modeled using an appropriate covariance structure to account for temporal correlations among measurements. Mean comparisons were performed using the least significant difference (LSD) test at *p* < 0.05.

Analyses of variance for WUE_WP_, biomass accumulation, and water use per plant were conducted using SAS 9.4. Relationships among WUE_WP_, δ^13^C, biomass accumulation, water use per plant, and F_V_/F_M_ across growth stages were assessed using Spearman’s rank correlation, which was selected because some variables did not fully satisfy the assumptions required for Pearson’s correlation. Pairwise trait relationships were visualized using scatterplot matrices, with histograms along the diagonal and correlation coefficients (r) and corresponding significance levels displayed in the upper panels. Linear regression lines were included to illustrate trait associations. Correlation analyses and graphical visualizations were conducted in R (Version 4.5.1; R Project for Statistical Computing; accessed 23 May 2026) using the GGally (Version 3.5.2; ggplot2 package website; accessed 23 May 2026) and ggplot2 packages (Version 2.4.0; GGally package website; accessed 23 May 2026). Similar approaches have been used to examine relationships among agronomic traits in wheat [110,111,112].

The Shannon Diversity Index was used to assess phenotypic diversity within the spring wheat panel. The Shannon Diversity Index was calculated as follows:

*H′* = −∑(Pi × lnPi) (5)

where Σ is the summation symbol, ln is the natural logarithm, and Pi is the proportion of the entire panel represented by cultivar i. The higher the value of *H*′, the greater the diversity of cultivars in the panel. A value of *H*′ = 0 indicates no diversity. The Shannon diversity index typically ranges from 1.5 to 3.5 and rarely exceeds 4.5 [113]. The maximum possible value depends on the number of cultivars in the panel. All diversity index analyses were performed using Microsoft Excel.

Hierarchical clustering heatmap analysis was performed to investigate phenotypic relationships among the 198 Canadian spring wheat cultivars based on δ^13^C, WUE_WP_, biomass accumulation, and water use per plant. Trait values were standardized using Z-score normalization to ensure comparability among variables measured on different scales. Cultivars and traits were clustered using Euclidean distance and Ward’s hierarchical agglomerative method [114,115], and the resulting relationships were visualized through dendrograms and heatmaps. In the heatmap, red and blue colors indicate standardized values above and below the population mean, respectively [116]. This multivariate approach enabled the identification of cultivar groups with similar physiological response patterns and highlighted associations among traits related to carbon assimilation, water-use strategies, and biomass production. The clustering structure also provided insight into the extent of phenotypic variation within the germplasm panel and facilitated the detection of contrasting adaptive responses to drought and heat stress among cultivars.

Principal component analysis (PCA) was conducted using standardized values of δ^13^C, WUE_WP_, biomass accumulation, and water use per plant to characterize multivariate relationships among wheat cultivars. Variables were standardized prior to analysis to eliminate differences in measurement scale. PCA was used to summarize overall phenotypic variation, identify the traits contributing most to cultivar differentiation, and visualize relationships among cultivars and traits in reduced-dimensional space. The first two principal components (PC1 and PC2) were used for graphical representation, with trait vectors indicating the direction and relative contribution of each variable to the observed variation [117,118]. Prior to statistical analysis, the assumptions underlying each model were evaluated. For ANOVA and repeated-measures ANOVA, residual normality was assessed using normal probability plots and the Shapiro–Wilk test, while homogeneity of variance was evaluated through residual-versus-predicted value plots and Levene’s test. For repeated-measures analyses, alternative covariance structures were compared, and the most appropriate structure was selected based on model fit criteria. When assumptions were not fully met, data were carefully examined, and non-parametric approaches, including Spearman’s rank correlation analysis, were employed where appropriate to ensure robust statistical inference (Table 3).

## 5. Conclusions

Projected climate change in western Canada, characterized by higher temperatures, more frequent heatwaves, and reduced water availability, threatens wheat production and emphasizes the need to improve water-use efficiency and heat tolerance in Canadian spring wheat. This study revealed substantial phenotypic and physiological variation among 198 historical and modern cultivars for WUE_WP_, δ^13^C, biomass accumulation, water use, and chlorophyll fluorescence under combined drought and heat stress. Although significant variation was present, WUE_WP_ and δ^13^C displayed narrower trait distributions and lower diversity relative to biomass and water use, indicating that phenotypic variability for these traits is comparatively restricted within the evaluated germplasm. The weak relationship between WUE_WP_ and δ^13^C indicates that carbon isotope composition alone is not a reliable predictor of WUE_WP_ under combined drought and heat stress. Multivariate analyses identified distinct physiological response patterns among cultivars, highlighting the complexity of stress adaptation. While F_V_/F_M_ was not directly associated with WUE_WP_ or δ^13^C, it showed relationships with biomass accumulation and water use during reproductive stress stages, supporting its value as a rapid indicator of physiological stress responses.

Overall, integrating physiological traits, chlorophyll fluorescence, and multivariate analyses provides an effective framework for evaluating wheat responses to drought and heat stress. The identification of cultivars with contrasting WUE_WP_, biomass accumulation, water use, and photosynthetic performance can support breeding efforts to improve climate resilience in western Canada. These findings also highlight the need to broaden the genetic base of Canadian spring wheat and adopt integrated multi-trait screening approaches rather than relying on individual traits or indicators.

## Figures and Tables

**Table 1:** Mean, minimum, maximum, standard deviation (SD), coefficient of variation (CV), and Shannon diversity index (*H′*) for whole-plant water-use efficiency (WUE), biomass, water use, and δ^13^C measured across 198 historical and modern wheat cultivars under controlled environmental conditions. Growth Chamber Run 1 and Growth Chamber Run 2 represent two independent growth-chamber runs conducted under identical environmental conditions, stress treatments, and experimental protocols.

Parameters	Mean	Max	Min	CV (%)	LSD_(0.05)_	Stdev	*p* > F	*H′*
	Growth Chamber Run 1							
δ^13^C (‰)	−26.37	−24.28	−29.33	-	-	0.92	-	2.62
WUE_wp_ (g L^−1^)	4.17	5.71	3.07	9.48	0.79	0.43	0.0003	1.88
Biomass (g)	16.10	23.85	9.50	12.13	3.89	3.36	<0.0001	1.57
Water use (L)	3.92	5.04	2.27	11.41	0.87	0.71	0.0269	1.43
	Growth Chamber Run 2							
δ^13^C (‰)	−26.00	−24.06	−28.16	-	-	0.80	-	2.49
WUE_wp_ (g L^−1^)	4.12	7.81	3.11	11.98	0.98	0.55	<0.0001	2.02
Biomass (g)	35.24	50.60	24.80	12.33	8.68	5.54	<0.0001	2.29
Water use (L)	8.73	12.34	3.17	18.42	3.21	1.31	0.34	2.15
	Growth Chamber Runs 1 and 2							
δ^13^C (‰)	−26.18	−24.06	-29.33	-	-	0.88	-	2.61
WUE_wp_ (g L^−1^)	4.15	7.81	3.07	11.95	1.18	0.57	0.0554	2.52
Biomass (g)	25.69	50.60	9.50	41.06	10.14	10.79	<0.0001	2.52
Water use (L)	6.30	12.34	2.27	41.95	2.6	2.57	<0.0001	1.98

**Table 2:** The breeding program origins of 198 historical and modern Canadian spring wheat cultivars registered in western Canada between 1905 and 2018 were used in the current study. CDC: Crop Development Centre; U of S: University of Sakatchewan; WPB: Wiersum Plant Breeding; CIMMYT: International Maize and Wheat Improvement Center; NDSU: North Dakota State University, USDA ID: United States Department of Agriculture; WFGD Co-op: Western Field Grain Development Cooperative registration trial; AAFC: Agriculture and Agr-Food Canada; U of A: University of Alberta; U of M: University of Manitoba.

Breeding	Number of	Cultivars					
Program	Cultivars						
AAFC	116	AACAwesome	AAC Iceberg	AC Abbey	Enchant	Peace	AC Barrie
		AAC Bailey	AAC Innova	AC Andrew	Fieldstar	Pembina	AC Cora
		AAC Brandon	AAC Jatharia	AC Cadillac	Garnet	PT472	AC Crystal
		AAC Cabri	AAC Penhold	AC Corinne	NRG097	PT479	AC Domain
		AAC Cameron	AAC Prevail	AC Intrepid	GoodeveVB	RL6077	AC Elsa
		AAC Castle	AAC Proclaim	AC Meena	Helios	Sadash	AC Foremost
		AAC Chiffon	AAC Raymore	AC Phil	HY320	Sinton	AC Karma
		AAC Cirrus	AAC Redwater	AC Snowbird	Kanata	Snowhite475	AC Majestic
		AAC Connery	AAC Ryley	AC Vista	Kane	Snowstar	AC Michael
		AAC Crossfield	AAC Spitefire	Alvena	Manitou	Stettler	AC Minto
		AAC Crusader	AAC Tenacious	Burnside	Marquis	Superb	AC Reed
		AAC Current	AAC Tradition	Canuck	Minnedosa	Unity	AC Splendor
		AAC Durafield	AAC Viewfield	Carberry	MuchMore	Waskada	AC Taber
		AAC Elie	AAC W1876	CDN Bison	Napayo	Whitehawk	Benito
		AAC Entice	AAC Whitefox	Conquer	Neepawa	Cardale	Biggar
		AAC Foray	AC 2000	Cypress	Park	AC Eatonia	Bluesky
		Bhishaj	SWS52	BW278	FL62R1	Cardale	
		Columbus	Lancer	Lillian	Somerset	Infinity	
		Grandin	Laura	Pasqua	Vesper	Lovitt	
		Katepwa	Leader	Roblin	Wildcat	Harvest	
CDC	31	CDC Carbide	CDC Kernen	CDC Stanley	CDC Fortitude	CDC Bradwell	CDC Titanium
U of S		CDC Abound	CDC Merlin	CDC Teal	CDC Cordon	CDC Go	
		CDC Alsask	CDC NRG003	CDC Thrive	CDC TERRAIN	CDC Hughes	
		CDC Imagine	CDC Osler	CDC Utmost	CDC VR Morris	CDC Bounty	
		CDC Walrus	CDC Whitewood	BW970	Conway	PT595	
		Kenyon	Moats	CDC Plentiful	CDC Primepurple	CDC Rama	
U of A	20	Alikat	Cutler	PT771	Ellerslie	Coleman	
		BW1039	Go Early	PT778	Tracker	Thorsby	
		BYT1411	GP168	PT780	Jake	BW493	
		BYT1419	Zealand	Laser	Parata	RedNet	
U of M	2	Amazon	Glenlea				
WPB	1	Pasteur					
Syngenta	14	5604HR CL	GP112	SY 433	SY637	SY985	
Canada Inc.		5605HR CL	SY087	SY479	5702PR	WR859 CL	
		5701PR	Invader	SY995	5700PR		
WFGD	1	WTF603					
NDSU	2	Faller	Prosper				
SK Wheat	4	Prodigy	McKenzie	Oslo	Journey		
CIMMYT	2	Pitic62	SAAR				
USDA ID	3	Owens	Springfield	Fielder			
Others	2	Red Bobs	Sumai3				

**Table 3:** Terms, formulas and description of the chlorophyll fluorescence parameters used in the study.

Parameter and Calculation	Description
PAR	Photosynthetically available radiation (PAR) represents the portion of solar radiation within the 400–700 nm wavelength range that is utilized by plants for photosynthesis (μmol m^−2^ s^−1^)
F_0_	Minimum chlorophyll *a* fluorescence yield measured in dark-adapted leaves when all PSII reaction centers are open.
F_V_ = F_M_ – F_0_	Variable fluorescence (F_v_) represents the difference between maximum fluorescence (FM) and minimum fluorescence (F_0_) in dark-adapted leaves and reflects the variable component of chlorophyll *a* fluorescence associated with photochemical activity of photosystem II (PSII).
F_M_	Maximum chlorophyll *a* fluorescence yield measured in dark-adapted leaves after application of a saturating light pulse.
F_V_/F_M_ = (F_M_ – F_0_)/F_M_	Maximum quantum yield of photochemistry in photosystem II (PSII) measured in the dark-adapted state (F_V_/F_M_) represents the maximum efficiency at which absorbed light energy can be converted into photochemical energy in PSII when all reaction centers are fully open. It is widely used as an indicator of photosynthetic performance and stress-induced damage to the photosynthetic apparatus.
F_V_/F_0_ = (F_M_ – F_0_)/F_0_	The $F_{V}/F_{0}$ ratio is commonly used as an indicator of the potential activity of PSII and the efficiency of the water-splitting complex on the donor side of PSII. Higher values generally indicate better photosynthetic performance and stress tolerance, whereas reductions often reflect damage or impairment of the photosynthetic apparatus under drought, heat, salinity, or other abiotic stresses.
F′_0′_ = F_0_/(F_V_/F_M_ + F_0_/F′_M_)	Minimum yield of Chl a fluorescence measured under ambient light. F′_0_ can be measured after a brief (∼ 1 s) period of darkness to promote opening of all reaction centers
F′_M_	Maximum yield of Chl a fluorescence measured under ambient light. F_o′_ can be measured after a brief (∼ 1 s) period of darkness to promote opening of all reaction centers
Y(II) = (F′_M_ − F′) /F′_M_	Effective quantum yield of photochemical energy conversion in PSII. 0Y(II) is a measuring protocol that was developed by Bernard Genty with the first publications in 1989 and 1990.
ETR = Y(II) × PAR × 0.84 × 0.5.	Relative electron transport rate, is the product of the effective photochemical yield of PSII, Φ_P_ = ΔF/F′_M_ = (F_M′_-F)/F′_M′_ and photosynthetic photon flux density (PPFD) [106,119]. Electron transport rate (ETR), estimated from chlorophyll fluorescence, is a widely used indicator of photosynthetic activity.
φPo = 1 − F_0_/F_M_	Maximum quantum yield of primary photochemistry at time zero (ΦP_0_ or φP_0_) represents the maximum efficiency with which absorbed light energy is converted into photochemical energy in photosystem II (PSII) when all reaction centers are fully open in the dark-adapted state. It reflects the potential efficiency of primary photochemistry in PSII.
φDo = F_0_/F_M_	Fraction of absorbed light energy dissipated as heat and fluorescence when PSII reaction centers are fully open. Quantum yield of energy dissipation at time zero in dark-adapted leaves.

## Data Availability

The original contributions presented in the study are included in the Supplementary Material. Further inquiries can be directed to the corresponding authors.

## Supplementary Materials

The following supporting information can be downloaded at: https://www.mdpi.com/article/10.3390/plants15131958/s1.

## Abbreviations

The following abbreviations are used in this manuscript:

ANOVA	Analysis of Variance
BBCH	Biologische Bundesanstalt, Bundessortenamt and Chemical industry scale (growth stage scale)
CF-IRMS	Continuous-Flow Isotope Ratio Mass Spectrometry
Chla	Chlorophyll *a*
CNHR	Canada Northern Hard Red
CPSR	Canada Prairie Spring Red
CPSW	Canada Prairie Spring White
CWAD	Canada Western Amber Durum
CWES	Canada Western Extra Strong
CWHWS	Canada Western Hard White Spring
CWRS	Canada Western Red Spring
CWSP	Canada Western Special Purpose
CWSWS	Canada Western Soft White Spring
F_V_	Variable Fluorescence
F_0_	Minimum Fluorescence Yield (dark-adapted state)
F_M_	Maximum Fluorescence Yield (dark-adapted state)
F_V_/F_M_	Maximum Quantum Efficiency of Photosystem II
H′	Shannon Diversity Index
IRMS	Isotope Ratio Mass Spectrometer
PAM	Pulse-Amplitude-Modulated (fluorometry)
PCA	Principal Component Analysis
PSII	Photosystem II
ΦPSI	Quantum Yield of Photosystem I
r	Pearson Correlation Coefficient
SWC	Soil Water Content
VPDB	Vienna Pee Dee Belemnite (carbon isotope standard)
WD	Water-Deficient (treatment)
WUE	Water Use Efficiency
WUE_WP_	Whole-Plant Water Use Efficiency
Carbon Isotope–Related Terms	
δ^13^C	Carbon Isotope Composition (relative difference in ^13^C/^12^C ratio)
R_a_	^13^C/^12^C Ratio of Atmospheric CO_2_
R_p_	^13^C/^12^C Ratio of Plant Material
Rsample	^13^C/^12^C Ratio of Sample
Rstd	^13^C/^12^C Ratio of Standard
Statistical/Mathematical Terms	
Ln	Natural Logarithm
Σ	Summation Symbol
Pi	Proportion of the *i*th genotype in the population
Units	
°C	Degrees Celsius
kPa	Kilopascal (pressure unit)
µmol m^−2^ s^−1^	Micromoles per square meter per second (light intensity)
g L^−1^	Grams per liter
g plant^−1^	Grams per plant
